# New Technologies in Coronary Artery Surgery

**DOI:** 10.5041/RMMJ.10118

**Published:** 2013-07-25

**Authors:** David Taggart, Rony-Reuven Nir, Gil Bolotin

**Affiliations:** 1 Professor of Cardiovascular Surgery, University of Oxford, England, UK;; 2 Cardiovascular Surgery, Rambam Health Care Campus and Technion Faculty of Medicine, Haifa, Israel and; 3 Director, Cardiovascular Surgery, Rambam Health Care Campus and Technion Faculty of Medicine, Haifa, Israel

**Keywords:** Coronary artery disease, external stent, minimally invasive grafting, multiple arterial conduits, off-pump coronary artery bypass

## Abstract

Coronary artery disease remains the leading cause of death in developed countries. Major recent studies such as SYNTAX and FREEDOM have confirmed that coronary artery bypass grafting (CABG) remains the gold standard treatment in terms of survival and freedom from myocardial infarction and the need for repeat revascularization. The current review explores the use of new technologies and future directions in coronary artery surgery, through 1) stressing the importance of multiple arterial conduits and especially the use of bilateral mammary artery; 2) discussing the advantages and disadvantages of off-pump coronary artery bypass; 3) presenting additional techniques, e.g. minimally invasive direct coronary artery bypass grafting, hybrid, and robotic-assisted CABG; and, finally, 4) debating a novel external stenting technique for saphenous vein grafts.

## MULTIPLE ARTERIAL CABG PROCEDURES

Late survival after coronary artery bypass grafting (CABG) is improved when the left internal mammary artery (LIMA) is grafted to the left anterior descending artery (LAD).^1^,^2^ LIMA has been recognized as the optimal conduit in CABG because of its superior patency rate and freedom from arteriosclerosis compared with the saphenous vein (SV).^3^ In anticipation of additional advantages with the use of a second arterial graft, surgeons currently use the right internal mammary artery (RIMA),^4^–^6^ radial artery (RA),^7^–^9^ or gastroepiploic artery as the bypass conduit.^10^ Several retrospective analyses have documented an incremental survival benefit by increasing the number of arterial grafts,^4^,^5^,^9^,^11^ and two independent meta-analyses have corroborated a long-term benefit.^5^,^12^ Despite this compelling information in the published literature, multiple arterial grafting (MultArt) is currently performed in < 13% of CABG operations.^13^

A recent observational, retrospective study^14^ reviewed 8,622 Mayo Clinic patients who had isolated primary coronary artery bypass graft surgery for multivessel coronary artery disease from 1993 to 2009. Patients were stratified by number of arterial grafts into the LIMA plus saphenous veins (LIMA–SV) group (*n* = 7,435) or the MultArt group (*n* = 1,187). Propensity score analysis matched 1,153 patients. Operative mortality was 0.8% (*n* = 10) in the MultArt and 2.1% (*n* = 154) in the LIMA–SV (*P* = 0.818 for the propensity-matched analysis). Late survival was greater for MultArt versus LIMA–SV (10- and 15-year survival rates were 84% and 71% versus 61% and 36%, respectively (*P* < 0.001), in unmatched groups and 83% and 70% versus 80% and 60%, respectively (*P* = 0.0025), in matched groups). MultArt subgroups with bilateral internal mammary artery (BIMA)–SV (*n* = 589) and BIMA only (*n* = 271) had improved 15-year survival (86% and 76%; 82% and 75% at 10 and 15 years, *P* < 0.001), and patients with BIMA–RA (*n* = 147) and LIMA–RA (*n* = 169) had greater 10-year survival (84% and 78%, *P* < 0.001) versus LIMA–SV. In multivariate analysis, MultArt grafts remained a strong independent predictor of survival (hazard ratio 0.79, 95% confidence interval 0.66–0.94, *P* = 0.007). These findings suggest that in patients undergoing isolated coronary artery bypass graft surgery with LIMA to left anterior descending artery, arterial grafting of the non-left anterior descending vessels conferred a survival advantage at 15 years compared with SV grafting. It is still unproven whether these results apply to higher-risk subgroups of patients.

Despite previous reports of greater benefit from left than right coronary system grafting with the second arterial graft,^4^,^15^ a careful review of the literature indicates that use of two internal mammary artery (IMA) grafts demonstrates excellent long-term results with no demonstrable difference in outcome between right and left coronary system patients.^16^,^17^ Indeed, in the study by Locker et al., 20% of MultArt patients received the second arterial bypass to the right system only, with no additional arterial grafting to the circumflex coronary system.^14^

## BILATERAL INTERNAL MAMMARY ARTERIES

Almost three decades ago, in a seminal study, the Cleveland Clinic Group reported that a single internal mammary artery (SIMA) resulted in superior survival benefit as well as a reduced subsequent incidence of myocardial infarction, recurrent angina, and the need for repeat revascularization.^1^ This improvement in survival has now been reported to persist into the second and third decades of follow-up.^4^,^6^,^18^ More than a decade ago our own group published a systematic review including a meta-analysis of 15,962 patients receiving SIMA or BIMA grafts. The hazard ratio for death with BIMA grafts was 0.81, with a 95% confidence interval of 0.70–0.94.^5^ Although this was not a randomized trial the patients were matched for age, gender, diabetes, and ventricular function, four factors which give a likely indication of longevity even independent of the presence of coronary artery disease.

The most likely explanation for the survival benefit of IMA grafting is its greatly superior rates of patency in comparison to vein grafts. Whereas 10 years after bypass grafting up to three-quarters of all vein grafts are occluded or severely diseased, in contrast the patency rates of IMA grafts remain in excess of 90% even into the second decade of follow-up.^5^

The IMA graft is a unique artery both in anatomical structural terms (with a high proportion of elastic rather than muscle or adventitia composition) and in physiological function (it produces much greater levels of nitric oxide and decreased release of vasoconstrictors in comparison to other vessels), resulting in potent anti-atherosclerotic effects.

Despite strong clinical evidence in favor of the use of BIMA grafts, their use in current practice remains disappointingly low, being around 5% of patients in the USA and fewer than 10% in Europe.

In an effort to add more scientific data to the debate of SIMA or BIMA grafting, the Arterial Revascularization Trial (ART) randomized 3,102 patients in 28 centers in seven countries.^19^ The 1-year outcomes showed 30-day mortalities of just over 1% in both groups and just over 2% at 1 year, with no significant difference in the incidence of stroke, myocardial infarction, and repeat revascularization (i.e. safety end-point), which were all around 2%. This clearly demonstrated that there was no increase in mortality or myocardial infarction with BIMA grafts. Furthermore the use of a second IMA graft added 23 minutes to the operative procedure which in itself took 3–4 hours.

The one note of caution was that there was indeed an increase in sternal wound reconstruction from 0.6% in the SIMA group to 1.9% in the BIMA group, i.e. an absolute difference of 1.3% or a number needed to harm of 78 patients. However, it is noteworthy that while one-quarter of all patients in the ART Trial had diabetes almost half the patients requiring sternal wound reconstruction had diabetes. It is highly likely that with more judicious patient selection (avoiding BIMA grafts in obese diabetics or those with impaired lung function) and more precise harvesting techniques (skeletonization rather than pedicle to preserve collateral circulation)^20^ the incidence of sternal wound reconstruction would be significantly lower.

While the results of recent trials of CABG versus stents in general populations (such as the SYNTAX Trial) and in diabetics (the FREEDOM Trial) confirm the significant superiority of CABG over stents in terms of superior survival and freedom from subsequent myocardial infarction or repeat revascularization, the low use of BIMA grafts in current practice is a poor reflection of optimal surgical therapy. The recommendations in guidelines support the use of more arterial grafts during CABG,^21^,^22^ and the National Societies of Cardiothoracic Surgery should give increased recognition to and promote more use of BIMA grafts.

## OFF-PUMP SURGERY

For almost three decades there has been controversy as to the potential benefits of off-pump CABG in relation to on-pump CABG. The initial rationale for off-pump CABG was mainly driven by economic considerations in developing countries where the economic cost of cardiopulmonary bypass made CABG an unrealistic proposition in many patients. Despite much skepticism by its opponents, off-pump CABG was gradually introduced into developed countries where its proponents recognized its potential to mitigate the adverse effects of extracorporeal circulation in an increasingly elderly population undergoing CABG. The views of supporters and opponents of off-pump CABG have remained essentially unchanged in the intervening period.

A meta-analysis by Afilalo and colleagues^23^ of almost 9,000 patients from 59 randomized trials showed no difference between the two techniques in postoperative mortality and myocardial infarction but did report a lower incidence of stroke in the off-pump group (1.4% versus 2.1%, odds ratio 0.7, 95% CI 0.49–0.99). However, an important consideration in many of the randomized trials was the question about the actual surgical experience of those performing the off-pump surgery. Indeed, two trials reporting worse outcomes with off-pump surgery were severely criticized on the basis of the inexperience of the participating surgeons—emphasized by high rates of conversion from off-pump to on-pump surgery.^23^,^24^

Two recently published trials provide far more definitive answers. First, the CORONARY Trial, which enrolled 4,752 patients in 79 centers in 19 countries, had previously reported no significant difference at 30 days in the primary composite outcome of death, myocardial infarction, stroke, or new renal failure between the two techniques.^25^ The trial has now reported the 1-year outcomes^26^ and showed no significant difference in the primary composite outcome between off-pump and on-pump CABG (12.1% off-pump versus 13.3% on-pump, hazard ratio 0.91,*P* = 0.24). In particular, there was no difference in the incidence of individual components of the primary outcome in terms of death, myocardial infarction, stroke, or new renal failure. Furthermore, and in contrast to previous studies, there was no significant increase in the incidence of repeat revascularization for off-pump CABG at 1 year. Additionally, there was no difference in neurocognitive outcomes at 1 year between the two groups. The most likely explanation of the differences between the findings of the CORONARY Trial and two of the largest previous trials reporting inferior outcomes for off-pump CABG is that the CORONARY Trial not only enrolled a far greater number of patients but, crucially, recruited surgeons with a far higher level of surgical expertise in off-pump surgery.

A second trial (GOPCABE), which randomized 2,539 patients aged 75 years or older to on-pump and off-pump CABG, has been published very recently.^27^ Again, the primary outcome was a composite of death, stroke, myocardial infarction, repeat revascularization, or new renal replacement therapy at 30 days and at 1 year after surgery. The authors reported no significant differences in the composite outcome either at 30 days (7.8% off-pump versus 8.2% on-pump, *P* = 0.74) or at 12 months (13.1% versus 14%, *P* = 0.48). Of particular note in this trial is the fact that the surgeons were highly experienced; for off-pump surgeons the average number of off-pump surgeries was 514 and for on-pump surgeons 1,378. Although the average number of coronary anastomoses was 2.7 in the off-pump group and 2.8 in the on-pump group (*P* < 0.001), this is highly unlikely to be of any clinical significance. The only remaining question now would appear to be whether off-pump surgery in association with a no-touch aortic technique significantly reduces the risk of perioperative stroke. It is noteworthy that in the GOPCABE Trial the most common reason for conversion from on-pump to off-pump CABG after the skin incision was a calcified ascending aorta.

In summary, the postulated benefits of off-pump surgery have not materialized in clinical practice for most patients, possibly due to the fact that advances in extracorporeal perfusion have made cardiopulmonary bypass much safer. For most patients undergoing CABG today the use of bilateral internal mammary arteries is far more important than whether surgery is performed on or off pump.

## MINIMALLY INVASIVE DIRECT CORONARY ARTERY BYPASS GRAFTING

Minimally invasive direct coronary artery bypass grafting (MIDCAB) utilizes a small anterior left thoracotomy incision and harvesting of the left internal mammary artery with an anastomosis performed to the left anterior descending artery without cardiopulmonary bypass. MIDCAB was initially described for single-vessel bypass to the left anterior descending (LAD) artery.^28^ Many variations have been described, including the single left internal mammary artery (LIMA) to LAD bypass, the multivessel complete revascularization, and the saphenous vein graft from the axillary artery to the LAD. Mammary harvest variations include robotic and thoracoscopic takedown. Finally, MIDCABs have been done with and without cardiopulmonary bypass (CPB).^29^

Patients for the MIDCAB approach are to be selected carefully; the ideal candidate would have severe stenosis or complete occlusion of the proximal LAD. It is imperative that the distal LAD is visualized either by collateral filling or by computed tomographic angiography in cases in which the patient has complete occlusion. Importantly, obesity is a relative contraindication; although the LIMA takedown is technically possible in obese patients, the pressure placed on the wound edges by the retractor can lead to tissue necrosis and wound infections. Similarly, female patients with large breasts are at increased risk of wound necrosis.^30^

The most pivotal factors in the postoperative management of MIDCAB patients are analgesia and early mobilization^30^; many patients are extubated on the table, but if a period of postoperative ventilator support is required, the endotracheal tube is changed to a single-lumen tube. Non-steroidal anti-inflammatory medications are used in addition to narcotics, and occasionally a thoracic epidural catheter is placed for pain control. Intravenous fluids are restricted, and patients are usually allowed to get out of bed the same evening. Monitoring lines and chest tubes are removed on the first postoperative day, and patients ambulate aggressively. Once pain is well controlled with oral medications, patients are discharged home usually on the third or fourth postoperative day.

The overall reported results of MIDCAB have been excellent,^31^-^35^ as: 1) Procedural success is estimated at 98%; 2) Operative mortality is < 1% in most series; 3) Reoperation rates for bleeding vary from 1% to 3%; 4) Chest wound complications occur in 2%–3%; 5) Pulmonary complications are seen in 1%–3% of patients; 6) Angiographic patency in the early postoperative period and at 6 months has been outstanding; and 7) Re-intervention for ischemic events has been atypical.

## HYBRID MIDCAB APPROACH

Recently, several studies reported a fruitful use of a hybrid approach combining minimally invasive LIMA–LAD bypass procedures with catheter-based interventions on the circumflex or right coronary arteries for the treatment of multivessel disease. In most series, the catheter-based interventions, which generally necessitate the placement of a drug-eluting stent, were performed several days before or several days after the surgical revascularization,^36^ although a same-day hybrid approach has also been described^37^; both methodologies suggest that integrated revascularization treatment plans provide minimally invasive options for patients with multivessel coronary artery disease.

A very recent study^38^ evaluated the long-term outcomes of minimally invasive hybrid revascularization based on a 13-year long database (1997–2011) of 810 MIDCAB procedures of isolated revascularization in 644 patients; MIDCAB, as a part of hybrid revascularization, was associated with percutaneous coronary intervention (PCI) in 166 patients. In line with previous reports, results indicated the following:1) Overall mortality: 0.24%; 2) Perioperative acute myocardial infarction: 1.6%; 3) Early reoperation: 0.74%; 4) Reopening for bleeding: 1.2%; 5) Case rate of hemotransfusion: 3.1%; and 6) Mean hospital postoperative stay: 4 ± 2.5 days. Postoperative angiographic control prior to PCI and in symptomatic patients showed patent left internal mammary artery in 100% of cases. Notably, in the hybrid revascularization group, at the mean follow-up of 4.5 ± 2.3 years, freedom from related cardiac death was 93% and freedom from cardiac re-intervention was 83%.

Theoretically, hybrid procedures provide a complete revascularization while keeping the survival benefit and angina relief of a LIMA–LAD graft and avoiding the morbidity of sternotomy.^39^ The ideal candidate for the hybrid approach may be a patient with double- or triple-vessel disease with low syntax score or a patient with high syntax score and high Euroscore. Before prevalent implementation of this approach will occur, however, patency and outcome data are required. Though more laborious and cost-intensive compared to traditional CABG or stenting, improvements in the techniques and co-ordination between the surgeon and interventional cardiologists will probably increase the effectiveness and value of the hybrid approach.^39^ A designated hybrid operative room will allow performing a single-session procedure at one place without the need to transfer the patient from the operating room to the catheterization laboratory.

## ROBOTIC-ASSISTED CABG

The surgical robot is an elegant microprocessor-controlled, electromechanical instrument that allows the surgeon to remotely manipulate fully articulating videoscopic instruments by way of master–slave servos and microprocessor control. These long, thin instruments, which can be inserted into the closed chest through half-inch incisions, are designed to allow multiple degrees of freedom and can precisely emulate the surgeon’s movements at the control console.^40^ A clear benefit to the robotic approach over other methods, however, has not been demonstrated.

Since the introduction of surgical robotics in the 1990s, there has been a progressive increase in utilization for thoracic surgical procedures. Although mitral valve and non-cardiac thoracic procedures account for the majority of cases, there are increasing reports of robotic-assisted coronary revascularization procedures. These reports include robotic LIMA harvest followed by a traditional MIDCAB^41^ or left thoracotomy off-pump CABG,^45^ totally endoscopic coronary artery bypass (TECAB) on the arrested heart,^42^,^43^ and totally endoscopic bypass without CPB (OP-TECAB).^43^ Although most TECABs and OP-TECABs involve only a LIMA–LAD graft, recent reports described a series of multivessel revascularization procedures.^42^ These series have demonstrated that each of these methods of limited access off-pump coronary bypass is associated with a shorter hospital stay, less time on mechanical ventilation, fewer transfusions, and a more rapid return to full activity. The operative times are considerably longer than for open procedures, but improved time efficiency with experience is the norm. Also, questions related to graft patency and long-term results persist. Several earlier reports suggested a conversion to an open procedure in > 50% of cases, but with increased experience conversion in the ≤10% range is more common.^43^

Because of the added expense and difficulty with learning the technique, the routine use of surgical robotics in CABG surgery does not seem likely in the near future. The robot has and will continue to evolve. Improved video resolution, lower-mass arms, the addition of a fourth tele-manipulator, and the availability of an elegant robotic coronary stabilizer will likely increase its effectiveness and extend its application. Refinement of automated distal anastomotic devices may further increase the growth of robotic coronary revascularization surgery.

## PATENCY OF GRAFT

Coronary artery disease (CAD) remains the leading cause of death in developed countries, and recent studies such as SYNTAX and FREEDOM have confirmed that CABG remains the gold standard treatment in terms of survival and freedom from myocardial infarction and the need for repeat revascularization. Despite strong evidence of an additional survival benefit of BIMA over a SIMA, only around 5%–10% of patients receive BIMA or additional arterial grafts. The saphenous vein graft (SVG) is still the most commonly used conduit because of its abundance, ease of harvest, and “user friendliness.” However, its main disadvantage is its relatively poor long-term patency compared to IMA grafts, with graft failure in as many as 20% of veins within the first year and in as many as 50% at 10 years and with further significant disease in half of the remaining patent grafts (in comparison to perfect patencies of 90%–95% of IMA grafts). SVG failure can result in major adverse clinical sequelae (including myocardial infarction, re-interventions, and death).

Vein graft failure appears to result from both medial and neo-intimal thickening, caused by migration and proliferation of smooth muscle cells and the late appearance of mature lipid-laden atherosclerotic plaques. These changes can compromise flow directly or promote thrombotic occlusion. Diffuse neo-intimal tissue proliferation, the origin of vein graft disease, develops in 75% of grafts within 1 year of implantation. This occurs because the vein graft is exposed to a “new” mechanical environment in the arterial circulation, with relatively high pressures and shear stress. In the first few weeks, shear-induced remodeling leads to luminal enlargement followed by a later phase typified by wall tension-induced remodeling leading to wall thickening (intimal hyperplasia) and stiffening. It is also believed that luminal irregularities of the native vein and its valves are additional triggers for aggressive focal intimal hyperplasia, further increasing the risk of vein graft failure. Neither antiplatelet therapy nor avoidance of surgical preparative injury has been shown conclusively to eliminate medial and neo-intimal thickening in either experimental models or human vein grafts.

## METHODS TO EXTEND SAPHENOUS VEIN GRAFT PATENCY

In addition to the clinically well-established ways of improving vein graft patency, such as a low-cholesterol diet and smoking cessation,^44^ new *in vitro* and *in vivo* experimental attempts have been made to reach the same pivotal goal. The employed experimental strategies include the use of 1) pharmacological agents, such as lidocaine, which was studied *in vitro* using standard tissue bath techniques^45^; 2) gene targeting, e.g. short interfering RNA (siRNA)-mediated silencing of adhesion molecule^46^; and two additional methods that are elaborated hereunder: 3) vein harvesting, and 4) external stents.

Various vein harvesting techniques were explored in order to extend the SVG patency, including the “no-touch” saphenous vein harvesting, which refers to the saphenous vein removal with minimal trauma and preservation of the normal architecture^51^; notably, the “no-touch” technique seems to produce a superior graft with long-term patency comparable to the internal thoracic artery. Moreover, reducing the distending pressure while harvesting the SVG was suggested to increase the SVG patency.^47^

## THE VENOUS EXTERNAL SUPPORT TRIAL (VEST)

Using an external stent to prevent vein graft dilation and mitigate luminal irregularities and wall tension has been hypothesized to reduce intimal hyperplasia and consequently vein graft failure. However, previous attempts at external stenting of vein grafts have failed for a variety of reasons. VGS FLUENT (RAD BioMed, Tel-Aviv, Israel), a novel external support device for SVGs, is a cobalt chrome braid, with a unique combination of different types of wires which provide it with axial plasticity (i.e. can stretch to cover the entire length of a vein graft) and radial elasticity (makes the vein graft crush- and kink-resistant while providing beneficial biomechanical properties by reducing wall tension and the diameter mismatch with the host artery). The stent maintains its position without any additional fixation such as using glue and can be applied in less than a minute without affecting current grafting technique.

**Figure 1:**
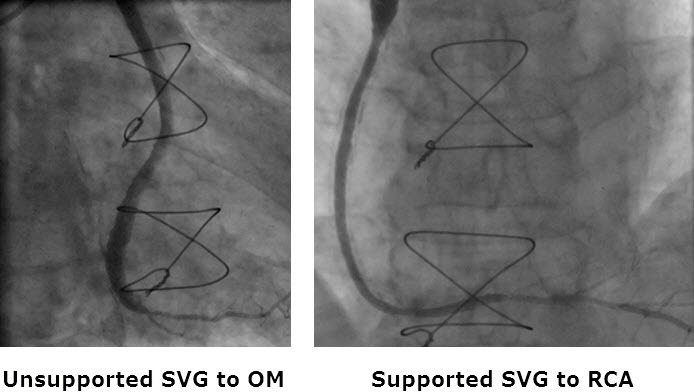
**Angiography 12 Months Post-CABG.** The unsupported vein graft show marked non-uniform expansion compared to the supported grafts.

**Figure 2:**
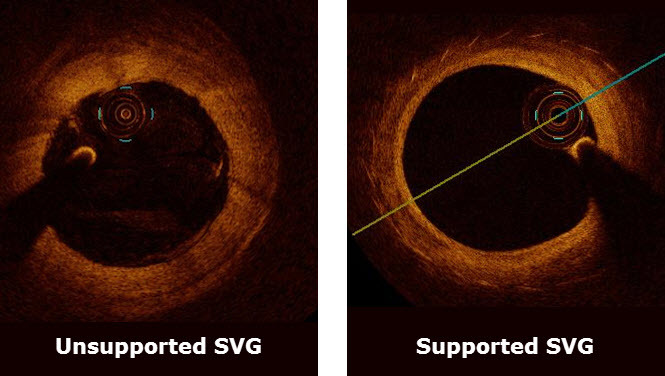
**Optical Coherence Tomography Cross-Sections of Vein Grafts 12 Months Post-CABG.** The supported vein graft has a thinner and more uniform intima layer.

A CABG study in sheep demonstrated the FLUENT’s safety along with excellent efficacy in reducing intimal hyperplasia, preventing vein graft dilation/deformation, and eliminating thrombus formation. Following these successful animal studies the FLUENT has been evaluated in a randomized controlled study (Venous External Support Trial) in the UK, which recruited 30 patients in Oxford and Brompton/Harefield who, in addition to an IMA graft to the LAD, required vein grafts to the right coronary artery and the circumflex artery. Patients were randomized for one vein graft to receive the stent and the other to act as a control. Patients are now undergoing 12-month-postprocedure angiography (Figure 1), intravascular ultrasound, and optical coherence tomography (Figure 2) to compare the experimental and control grafts’ patency, lumen uniformity, and plaque volume (intimal and medial hyperplasia). If the VEST successfully reproduces the findings in the sheep model, then the VEST investigators plan to undertake a multicenter trial in Europe, including several UK centers. If the stent is successful in significantly reducing intimal hyperplasia, it will undoubtedly become a “game changer.”

## Abbreviations

BIMA: bilateral internal mammary artery;
CABG: coronary artery bypass grafting;
CAD: coronary artery disease;
CPB: cardiopulmonary bypass;
IMA: internal mammary artery;
LAD: left anterior descending;
LIMA: left internal mammary artery;
LIMA–SV: LIMA plus saphenous veins;
MIDCAB: minimally invasive direct coronary artery bypass grafting;
MultArt: multiple arterial grafting;
PCI: percutaneous coronary intervention;
RA: radial artery;
RIMA: right internal mammary artery;
SV: saphenous vein;
SVG: saphenous vein grafts;
TECAB: totally endoscopic coronary artery bypass.
